# Backtracking to the Brain: A Journey From Cirrhosis to Hypothalamic Insight

**DOI:** 10.7759/cureus.84644

**Published:** 2025-05-22

**Authors:** Holly N Haley, Farukh G Ikram, Anne M Ward

**Affiliations:** 1 Internal Medicine, Methodist Dallas Medical Center, Dallas, USA; 2 The Liver Institute, Methodist Dallas Medical Center, Dallas, USA

**Keywords:** cryptogenic cirrhosis, growth hormone deficiency, hypothalamic mass, insulin-like growth factor-1 deficiency, panhypopituitarism, thyroid hormone receptor beta

## Abstract

We present a 31-year-old female patient with cryptogenic cirrhosis admitted for rectal bleeding secondary to rectal prolapse. During her hospital course, she was found to have severe multi-endocrine dysfunction as evidenced by persistent hypotension, bradycardia, and intermittent hypothermia with hypothyroidism, adrenal insufficiency, and diabetes insipidus, which were confirmed by laboratory testing. Due to the patient’s hypothalamic-pituitary dysfunction along with biopsy-confirmed cirrhosis of unknown etiology, magnetic resonance imaging (MRI) of the brain was ordered. The MRI demonstrated a large, heterogeneously enhancing mass centered in the hypothalamus and infiltrating the pituitary stalk. This case stands out because of its diagnostic trajectory where investigating the cause of endocrine dysfunction revealed a cerebral neoplasm that contributed to the patient’s development of cirrhosis. There is an established association between hepatic pathologies and hypothalamic masses with the proposed mechanism being deficiencies of growth hormone (GH), insulin-like growth factor-1 (IGF-1), thyroid stimulating hormone (TSH), and consequently, triiodothyronine (T3). GH deficiency predisposes patients to hepatic steatosis while IGF-1 and T3 deficiencies leave the liver more vulnerable to oxidative damage. As such, cranial imaging and endocrine evaluation should be considered in young patients with cryptogenic cirrhosis.

## Introduction

While common etiologies of end-stage liver disease include chronic alcohol use, metabolic disease, viral infection, autoimmune processes, and toxin ingestion, there is a subset of patients diagnosed with cryptogenic cirrhosis, in which there is no obvious driving force despite extensive workup [1]. For this reason, recent studies have evaluated additional systemic contributors to hepatic injury, specifically processes related to endocrine dysfunction. Among these, growth hormone (GH), insulin-like growth factor 1 (IGF-1), and thyroid hormone receptor-β (THR-β) have emerged as central regulators of hepatic function, metabolism, and repair [2-4]. In the setting of intracranial pathology, disruption within these hormonal axes plays a critical role in the pathogenesis of liver injury [5]. This case presents a young patient with cirrhosis of unknown etiology who was eventually found to have a hypothalamic mass, contributing to the development of accelerated hepatic disease.

This case was previously presented as a poster at the Texas Chapter of the American College of Physicians Annual Meeting on November 17, 2024, and at the American College of Physicians Annual Meeting on April 4, 2025.

## Case presentation

A 31-year-old female patient with a past medical history of cryptogenic cirrhosis of the liver, chronic constipation, anemia, and hypothyroidism was admitted for evaluation of rectal bleeding due to rectal prolapse and an initial hemoglobin level of 4.7 g/dL (reference range: 12.0-16.0 g/dL).

Two years prior to this admission, the patient had been diagnosed with hepatic cirrhosis during an investigation of a previous gastrointestinal bleed thought to have originated from the anastomosis of her resection rectopexy. The patient had followed up regularly with a hepatology clinic and had undergone extensive diagnostic workup for her liver including biopsy, magnetic resonance elastography, and magnetic resonance cholangiopancreatography. There was no evidence of steatosis or autoimmune, genetic, alcoholic, or infectious etiologies. Mild hepatic iron deposition was observed on pathology obtained from the liver biopsy, but subsequent testing was not consistent with hemochromatosis. Her alpha-fetoprotein level was minimally elevated at 7.71 IU/mL. Other mild elevations were noted in angiotensin-converting enzyme (94 U/L), alpha-1-antitrypsin (233 mg/dL), and ceruloplasmin (46 mg/dL). Interestingly, at the time of diagnosis, her C-reactive protein (CRP) level was significantly elevated at 206 mg/L with an erythrocyte sedimentation rate (ESR) of 41 mm/h. Additional laboratory tests that were within normal limits included: antinuclear antibody, antimitochondrial antibody, anti-smooth muscle antibody, liver-kidney microsomal antibody, anticentromere antibody, immunoglobulins A/E/G/M, and both chronic and acute viral hepatitis testing (Table 1).

**Table 1:** Previous hepatology workup

Laboratory test	Result	Reference range
Hereditary hemochromatosis DNA analysis	Wild-type	Wild-type (no mutations of HFE C282Y, H63D, and S65C)
Alpha-fetoprotein	7.71 IU/mL	<7.22 IU/mL
Angiotensin-converting enzyme	94 U/L	14-82 U/L
Alpha-1-antitrypsin	233 mg/dL	100-188 mg/dL
Ceruloplasmin	46 mg/dL	19.0-39.0 mg/dL
C-reactive protein	206 mg/L	<10 mg/L
Erythrocyte sedimentation rate	41 mm/h	0-20 mm/h
Antinuclear antibody	Negative	Negative
Antimitochondrial antibody	<20.0 units	<20.0 units
Anti-smooth muscle antibody	7 units	0-19 units
Liver-kidney microsomal antibody	1.6 units	<20.0 units
Anticentromere antibody	<0.2 AI	0.0-0.9 AI
Immunoglobulin A	118 mg/dL	87-352 mg/dL
Immunoglobulin E	51 IU/mL	6-495 IU/mL
Immunoglobulin G	1,227 mg/dL	586-1,602 mg/dL
Immunoglobulin M	83 mg/dL	26-217 mg/dL
Hepatitis A antibodies	Negative	Negative
Hepatitis B surface antibody	Reactive	> 10 mIU/mL consistent with immunity
Hepatitis B surface antigen	Negative	Negative
Hepatitis B core antibodies	Negative	Negative
Hepatitis C antibody	<0.1 S/Co	0.0-0.9 S/Co

The patient had no evidence of varices but did have trace ascites with splenomegaly. She was started on ursodiol for possible antimitochondrial antibody-negative primary biliary cholangitis with the intention of slowing disease progression. Her clinical course was complicated by admissions for hepatic encephalopathy, for which she was taking rifaximin.

At the time of her current hospitalization, her home medications were 325 mg ferrous sulfate daily, 137 µg levothyroxine daily, 550 mg rifaximin twice daily, 300 mg ursodiol twice daily, and furosemide and spironolactone as needed. Her past surgical history was significant for rectopexy, colonoscopy, and liver biopsy. The patient denied any history of alcohol or tobacco use, and notable family history was limited to melanoma in her father and type II diabetes mellitus in her mother.

On initial evaluation, the patient’s vitals were as follows: temperature of 35.2°C (95.3°F), heart rate of 72 beats per minute (bpm), blood pressure of 91/41 mmHg, respiratory rate of 12 breaths per minute, oxygen saturation of 97% on room air, and a body mass index of 33 kg/m2. She appeared pale and lethargic. There was no active rectal hemorrhage; therefore, she did not require acute interventions beyond resuscitation with packed red blood cell transfusions.

However, after adequate resuscitation, the patient remained hypothermic, hypotensive, and bradycardic despite a stable hemoglobin level. As a result, further workup was initiated including thyroid hormone testing and cortisol levels. The patient was found to have low TSH (0.08 uIU/mL) (Table 2), free thyroxine (T4) (0.37 ng/dL), and triiodothyronine (T3) (1.46 pg/dL) levels (Table 3). At home, the patient was non-adherent with her levothyroxine regimen. Due to the inappropriately low TSH level in the absence of exogenous steroid use, the patient was diagnosed with central hypothyroidism. Her baseline cortisol and serum adrenocorticotropic hormone (ACTH) levels were decreased at 3.7 µg/dL and 4.8 pg/mL, respectively. After ACTH stimulation testing, her cortisol levels appropriately increased to 105 µg/dL at 30 minutes and 81.8 µg/dL at one hour. As such, she was also diagnosed with central adrenal insufficiency. The patient’s serum follicle-stimulating hormone (FSH), luteinizing hormone (LH), and IGF-1 levels were also low at 0.8 IU/L, <0.1 IU/L, and 17 ng/mL, respectively.

**Table 2:** Diagnostic evaluation of endocrine abnormalities, pituitary hormones TSH: thyroid-stimulating hormone, ACTH: adrenocorticotropic hormone, FSH: follicle-stimulating hormone, LH: luteinizing hormone, GH: growth hormone, ADH: antidiuretic hormone

Pituitary hormone	Patient results	Reference range
TSH	0.08 mIU/mL	0.45-4.50 mIU/mL
ACTH	4.8 pg/mL	7.2-63.3 pg/mL
FSH	0.8 IU/L	4.7-21.5 IU/L
LH	<0.1 IU/L	14.0-95.6 IU/L
GH	N/A	N/A
ADH	N/A	N/A

**Table 3:** Diagnostic evaluation of endocrine abnormalities, peripheral hormones T4: thyroxine, T3 triiodothyronine, ACTH: adrenocorticotropic hormone, IGF-1: insulin-like growth factor 1

Peripheral hormone	Patient results	Reference range
Free T4	0.37 ng/dL	0.78-2.19 ng/dL
Free T3	1.46 pg/dL	2.77-5.27 pg/mL
AM cortisol	3.7 µg/dL	4.3-22.4 µg/dL
ACTH stimulation test		
Cortisol at 30 minutes	105 µg/dL	Peak response > 18 µg/dL
Cortisol at 60 minutes	81.8 µg/dL	
Estradiol	<10 pg/mL	38.0-649.0 pg/mL
Testosterone	<4.3 ng/dL	15-70 ng/dL
IGF-1	17 ng/mL	87-286 ng/mL
Serum sodium	152 mmol/L	135-148 mmol/L
Urine osmolality	153 mOsm/kg H2O	>600 mOsm/kg H2O

**Figure 1:**
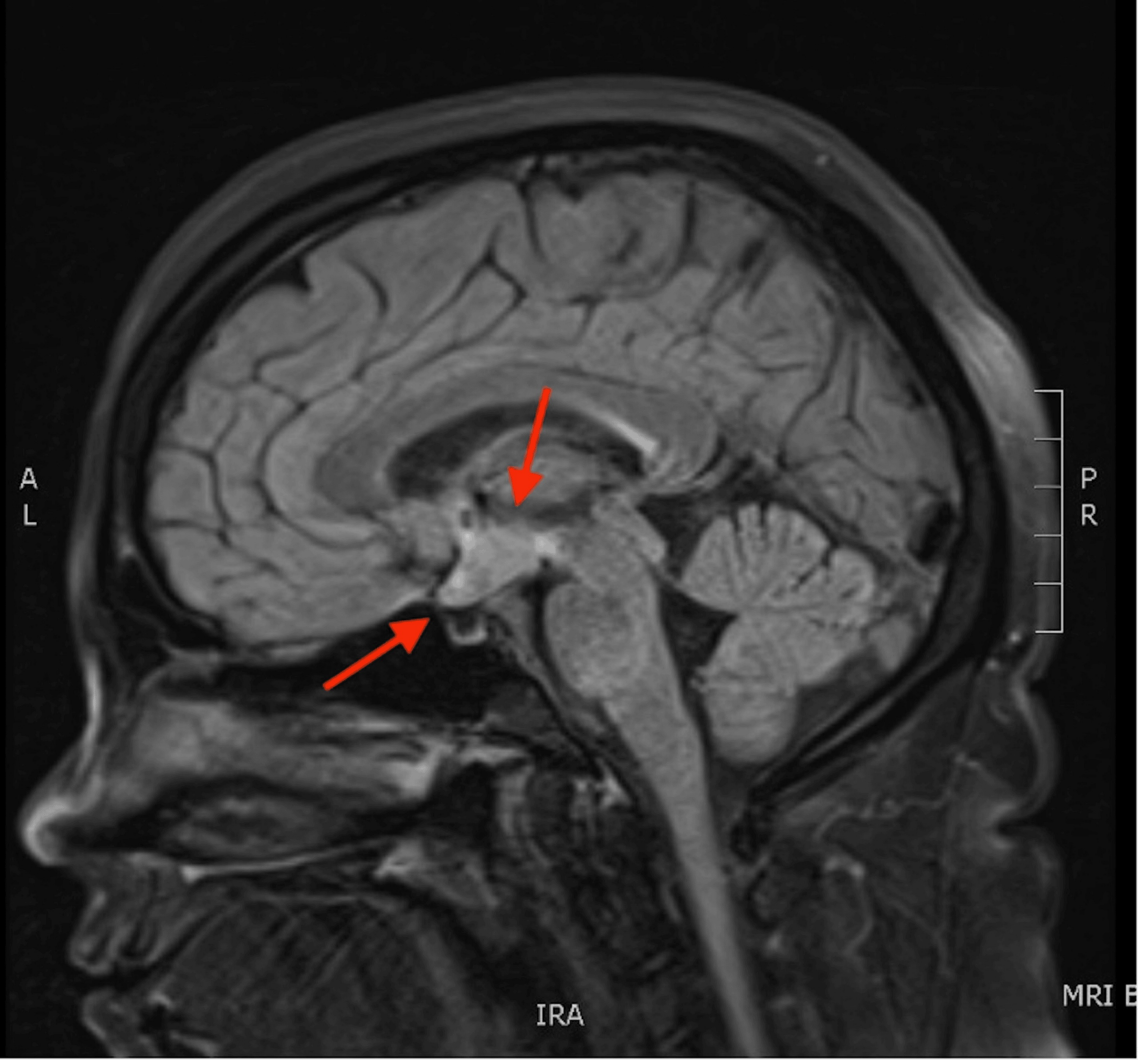
Magnetic resonance imaging in the sagittal plane demonstrating an enhancing hypothalamic mass measuring 2.4 cm x 1.7 cm x 1 cm with thickening of the optic chiasm.

 As part of the diagnostic workup for the patient’s hypothalamic-pituitary dysfunction, an MRI of the brain with and without contrast was performed. The images demonstrated an enhancing hypothalamic mass measuring 2.4 cm x 1.7 cm x 1 cm with thickening of the optic chiasm (Figure 1).

The pituitary gland was unremarkable with a tumor present only on the pituitary stalk. 

The patient had a witnessed aspiration event prior to her admission and required 3 to 4 L/min of supplemental oxygen. A computed tomography scan of the chest demonstrated nodular infiltrates on the right and minimal ground-glass opacities. As such, it was thought that she had aspiration pneumonitis which was present on admission. However, on hospital day seven, the patient’s respiratory status deteriorated, and she was in respiratory distress, desaturating to 60% after removing her oxygen delivery systems secondary to worsening mentation and agitation. Extensive bilateral pulmonary infiltrates were evident on repeat chest x-rays, and she was subsequently intubated and started on empiric intravenous antibiotic therapy. At this time she was hypothermic at 30°C (86°F), bradycardic to thirty (bpm) with first-degree atrioventricular block, and hypotensive at 76/46 mmHg. The patient was started on stress dose steroids and intravenous levothyroxine with improvement in her hemodynamic markers. Therapy with twice daily 0.5 µg intravenous desmopressin was also initiated for central diabetes insipidus due to polyuria, hypernatremia (152 mmol/L), and decreased urine osmolality. After developing acute respiratory distress syndrome, the patient had a complex and lengthy hospital course. She underwent a diagnostic bronchoscopy, which was unrevealing. Initial extubation failed due to tracheal edema and negative pressure pulmonary edema requiring reintubation. Hepatic cirrhosis and protein-calorie malnutrition predisposed the patient to ventilator dependence ultimately necessitating placement of a tracheostomy. She was discharged to a long-term acute care facility where she continued to improve. Outpatient follow-up with neurosurgery and endocrinology were arranged for the patient.

## Discussion

Though an uncommon occurrence, there have been reports of cirrhosis thought to be secondary to a mass in the hypothalamus [6-8]. This case is particularly unique in that the diagnosis of cirrhosis preceded identification of the neoplasm. As a direct consequence of her hypothalamic mass with involvement of the pituitary stalk, this patient effectively suffered from panhypopituitarism as evidenced by alterations in both anterior and posterior pituitary hormones. Although her prolactin level was within normal limits, she had decreased production of ACTH, TSH, FSH/LH, and likely GH from the anterior pituitary. ADH and oxytocin from the posterior pituitary were not directly measured, but the patient clinically presented with central diabetes insipidus given her hypernatremia and low urine osmolality, which were corrected with desmopressin.

Due to the pulsatile release of GH, IGF-1, which is produced in the liver, is often measured instead. The patient had decreased levels of IGF-1 with otherwise normal synthetic function of the liver; therefore, it is reasonable to assume that she was deficient in GH [8]. A deficiency of IGF-1, as seen in this case, is hypothesized to contribute to chronic hepatic disease via progressive inflammation [5,9]. The development of liver disease is often preceded by metabolic dysfunction, resulting in the accumulation of free fatty acids within hepatocytes. The formal insult occurs via the direct cytotoxic effects of free fatty acids, leading to oxidative stress from the reactive oxygen species generated by defective mitochondria [10]. While GH deficiency predisposes patients to hepatic steatosis, IGF-1 deficiency leaves the liver more vulnerable to oxidative damage [11]. Consequently, inflammatory cytokines such as tumor necrosis factor-alpha, which are catalyzed by lipotoxic species, cause hepatic stellate cells to transition from producing type IV collagen to synthesizing types I and III collagens, resulting in fibrosis [9,10]. Biopsy findings consistent with this mechanism include decreased glutathione and glutathione peroxidase in hepatocytes with an increased ratio of oxidized glutathione to glutathione [12]. Although these exact values were not measured in this patient, other inflammatory markers (e.g., CRP and ESR) were consistently elevated. Interestingly, studies have suggested that antioxidant activity observed on liver biopsy can be used as a prognostic factor in hepatic disease [12]. 

Thyroid hormones have also been shown to influence hepatic function, as thyroid hormone receptor-β (THR-β), which binds T3, is most prevalent in the liver and is important in reducing serum triglycerides [10]. T3 promotes mitochondrial oxidation through a combination of mitochondrial biogenesis and mitophagy [10]. This process increases the β-oxidation of fatty acids, ultimately leading to decreased lipotoxicity, oxidative stress, and inflammation [10]. Impairment of thyroid hormone signaling processes results in a cycle of repetitive liver injury. With chronic hepatic disease, there is decreased conversion of T4 to T3 by types I and II iodothyronine deiodinase (DIO1 and DIO2), and instead, there is an increased conversion of T4 to inactive reverse T3 by type III iodothyronine deiodinase. This shift favors the accumulation of lipotoxic species [10]. 

These processes are mediated by the complex interplay between GH and thyroid hormones as DIO1 and DIO2 are stimulated by GH. Therefore, GH deficiency is associated with decreased conversion of T4 to T3, and thus, decreased activation of THR-β [13]. In patients with a baseline deficiency of both GH and TSH, there is a significant reduction in the conversion from T4 to T3 when compared to someone with an isolated GH deficiency. Ongoing investigation is currently underway regarding IGF-1 and THR-β agonists as therapeutic options [9,10].

## Conclusions

This case indicates the importance of strongly considering cranial imaging and endocrine hormone testing in patients with cirrhosis where the underlying cause of liver damage is unknown, and when the degree of fibrosis is in excess of what would be expected at the patient’s age at presentation. It also sheds light on the potential importance of IGF-1 and emphasizes that although IGF-1 is a marker of cirrhosis, the hormone is protective for the liver. Deficiency of the hormone at baseline can predispose patients to the future development of cirrhosis. Additionally, the role of thyroid hormone signaling in the progression of hepatic injury as well as the intricate feedback amongst major endocrine axes is the current focus of many clinical trials targeting the management of non-alcoholic cirrhosis. Previous studies have observed the development of liver disease in patients with known hypothalamic tumors. However, this patient demonstrates that the reverse may also be true, and that the diagnosis of cirrhosis can precede identification of the brain mass.
